# Knowledge and Beliefs Associated with Environmental Health Literacy: A Case Study Focused on Toxic Metals Contamination of Well Water

**DOI:** 10.3390/ijerph18179298

**Published:** 2021-09-03

**Authors:** Kathleen M. Gray, Victoria Triana, Marti Lindsey, Benjamin Richmond, Anna Goodman Hoover, Chris Wiesen

**Affiliations:** 1 Institute for the Environment, University of North Carolina at Chapel Hill, Chapel Hill, NC 27599, USA; vtriana@email.unc.edu; 2 Southwest Environmental Health Sciences Center, University of Arizona, Tucson, AZ 85721, USA; lindsey@pharmacy.arizona.edu (M.L.); richmond@pharmacy.arizona.edu (B.R.); 3 Department of Preventive Medicine & Environmental Health, University of Kentucky, Lexington, KY 40536, USA; anna.hoover@uky.edu; 4 Odum Institute for Research in Social Science, University of North Carolina at Chapel Hill, Chapel Hill, NC 27599, USA; chris_wiesen@unc.edu

**Keywords:** environmental health literacy, drinking water, well water, toxic metals, self-efficacy

## Abstract

Environmental health literacy (EHL) is developing as a framework that can inform educational interventions designed to facilitate individual and collective action to protect health, yet EHL measurement poses several challenges. While some studies have measured environmental health knowledge resulting from interventions, few have incorporated skills and self-efficacy. In this study, a process-focused EHL instrument was developed, using the Newest Vital Sign (NVS) health literacy instrument as a model and tailoring it for the context of private well contamination with toxic metals. Forty-seven (47) participants, including undergraduate students and residents of communities with contaminated well water, piloted a prototype EHL instrument alongside NVS. Results suggested a moderate degree of correlation between NVS and the EHL prototype, and significant differences in scores were observed between students and residents. Responses to a self-efficacy survey, tailored for drinking water contaminated with arsenic, revealed significant differences between students and residents on items related to cost and distance. In response to open-ended questions, participants identified a range of potential environmental contaminants in drinking water and deemed varied information sources as reliable. This study highlights differences in knowledge and self-efficacy among students and residents and raises questions about the adequacy of EHL assessments that mimic formal education approaches.

## 1. Introduction

Over 42.5 million people in the United States get their drinking water from private wells [1], and this population has the potential to be exposed to elevated concentrations of arsenic and other toxic metals [2,3]. Such contamination may be undetected because private wells are not covered by the Safe Drinking Water Act (42 U.S.C. §§300f-300j-26). The lack of regulation underscores the importance to well users of understanding how poor well water quality can harm health, how to take action to identify and remove contaminants, and how to advocate for health-protective policies.

Environmental health literacy (EHL) provides a framework that can inform dialogue and interventions focused on well water quality, potentially leading to individual and collective actions that protect health [4]. Broadly, EHL has been defined as an understanding of how environmental exposures influence human health, including skills associated with finding and applying information to decisions about health risk [5,6]. Further, specific content knowledge, a core set of skills, and positive perceptions of self-efficacy related to health promoting behaviors have been identified as broadly underpinning EHL [7]. This multi-faceted definition recognizes that EHL is contextually dependent, with context including specific exposures and whether they occur at the individual or community level, among other factors. In the context of well water contamination, for instance, the EHL of well users could encompass such factors as understanding potential exposures, how such exposures may lead to adverse health impacts, and options for reducing or eliminating an exposure, along with development of skills related to sample collection or interpretation of well test results. The ability to implement actions that could reduce or prevent exposure, such as filter installation and maintenance, also is integral to EHL, as are individual beliefs about such abilities.

For a specific action, the concept of self-efficacy refers to an individual’s judgment of their capability to execute the action (such as the ability to test water for contaminants); the related concept of outcome expectancy (or perceived response efficacy in health communication) refers to beliefs about the likelihood that a desired outcome will result from those actions [8,9]. For example, science teachers who have confidence in their teaching abilities (self-efficacy) and believe that student learning can be influenced by effective teaching (outcome expectancy) are more likely to persist in their efforts [10]. Similarly, residents who have confidence in their ability to test their well water and believe that improved water quality is influenced by well testing may be more likely to engage in testing and persist in their efforts to implement health protective actions (such as choosing appropriate water treatment). The complexity of this interplay may be one reason that early EHL interventions tended to prioritize environmental health knowledge over skills and self-efficacy [4].

In recent years, a more robust set of instruments designed to assess EHL has been developed. Davis et al. [11] assessed the impact of a participatory training focused on rainwater harvesting and water contamination, finding that participants demonstrated increased knowledge and self-efficacy post-training. They also demonstrated increases in specific environmental health skills. Lichtveld et al. [12] developed and validated a survey that included scales for general EHL and media-specific (e.g., air, food, water) EHL. Each scale incorporated items that addressed knowledge, attitudes, and behaviors; and the authors asserted that the instrument could serve as a model for similar scales in additional environmental health domains. Specific to well water, Irvin et al. [13] developed a process-focused tool for assessing water-related EHL, titled Water Environmental Literacy Level Scale (WELLS) and reported positive associations between education, income level, and EHL. The tool effectively identified users with high and low EHL. Munene et al. [14] explored well users’ decisions to conduct testing by framing this environmental behavior as a health behavior. Though not an EHL study, their findings highlighted the role of self-efficacy in influencing behavior, along with well users’ perceived susceptibility to water contamination, perceived severity of the consequences of contamination, and the benefits of and barriers to well testing.

This study of EHL associated with toxic metals contamination of well water was conducted with residents in North Carolina (NC) and Arizona (AZ). In both states, a sizable percentage of the population has groundwater as the source of its drinking water [15,16]. Researchers associated with NIEHS-funded Environmental Health Sciences Core Centers are exploring the ways that arsenic impacts health, especially when ingested as drinking water [17,18]. Additionally, both states are home to racial/ethnic minority and rural populations, who are especially likely to experience health disparities related to contaminants in unsafe drinking water [19]. Thus, toxic metals contamination of groundwater is a relevant context for these Centers to explore residents’ EHL and effective methods of measuring it.

The impetus for this study and its emphasis on process-focused measurement of EHL associated with private well contamination was a desire by university researchers and educators to communicate more effectively with well users about the potential health impacts of such contamination. Understanding baseline levels of EHL is critical for developing educational materials that assist residents with testing wells and examining remediation options, as well as supporting well users in identifying health protective actions. In communities in NC, AZ, and Kentucky, the authors have participated in projects that aim to assist residents in identifying and remedying drinking water contamination, with a particular focus on arsenic exposure in well water in NC and AZ. In this context, the team wanted to better understand participants’ EHL to facilitate development of educational activities that were appropriately tailored. For this study, the research questions included the following: (a) How did health literacy scores from a validated, process-focused instrument compare to EHL scores from a prototype, process-focused instrument? (b) How did participants demonstrate environmental health knowledge and self-efficacy beliefs during formal assessments; and (c) Which variables, if any, included in an adapted self-efficacy scale influenced participant self-efficacy scores?

## 2. Materials and Methods

This study was conducted in NC, in a total of four research sessions. Two were convened on the campus of a large public university, and two were convened in communities that had identified concerns about toxic metals contamination of the private wells on which they relied for drinking water. Participants included (a) English-speaking undergraduate students majoring in disciplines other than STEM at a large public university and (b) English-speaking residents who obtain their drinking water from wells in communities with documented toxic metals contamination of well water. Convenience samples were recruited using email, listservs, and in-person gatherings of community-based organizations. The UNC-Chapel Hill Institutional Review Board reviewed all study protocols, and the study was deemed to be exempt (IRB#19-1103). Informed consent was obtained from all study participants.

The sample size was 47. Approximately 57% (*n* = 27) identified as female, approximately 57% (*n* = 27) identified as white, and approximately half reported living in rural areas. Participant ages ranged from 18 to 77, with a median age of 24 (Table A1 in Appendix A presents demographics of the sample.)

### 2.1. EHL Instrument

The instrument developed for this study (referred to as WEHL, for Water EHL) was designed to assess EHL in the context of contaminated well water and was modeled after a validated, process-focused health literacy instrument, The Newest Vital Sign (NVS) [20]. With NVS, health literacy is assessed based on patient responses to questions associated with a nutrition label, and the instrument was found to be effective as a quick screening test for limited literacy in health care settings [20]. NVS has been validated with varied populations, particularly younger adults and older English- and Spanish-speaking patients [21,22].

The WEHL incorporated essential environmental health knowledge and skills identified in prior research [7] and was formatted as a scenario in which a family dealing with health issues was deciding whether to consume or use groundwater with detectable arsenic. The WEHL included several components: (a) the family scenario; (b) two supporting artifacts (i.e., a hypothetical well water report and a handout with general information about arsenic and associated health effects); and (c) a series of questions about actions the family could take, as well as participant suggestions for seeking additional information. These components were designed to incorporate content knowledge specific to toxic metals exposure in groundwater, as well as artifacts that required reading and interpretation skills (i.e., general literacy, reasoning, and the ability to use numbers). Participants also completed a self-efficacy survey [23], which was tailored to assess the information-seeking domain for remedying contamination. Taken together, these components were designed to assess the knowledge and skills that contribute to EHL.

**Figure 1:**
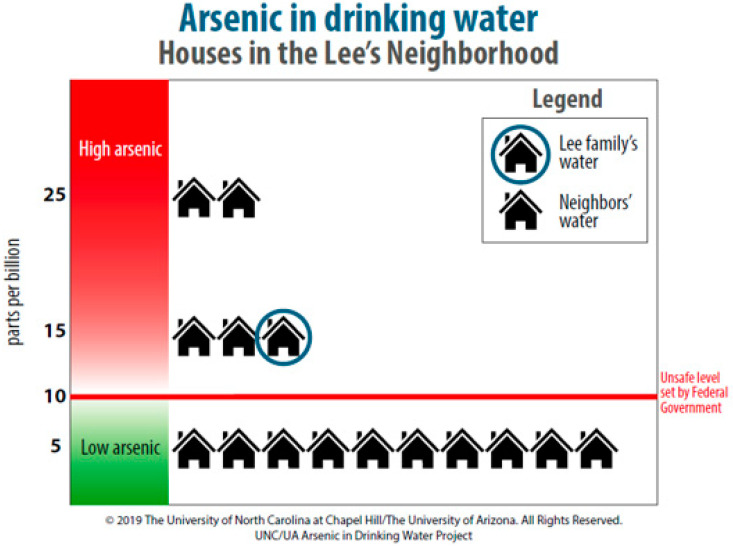
Hypothetical Well Water Report.

**Figure 2:**
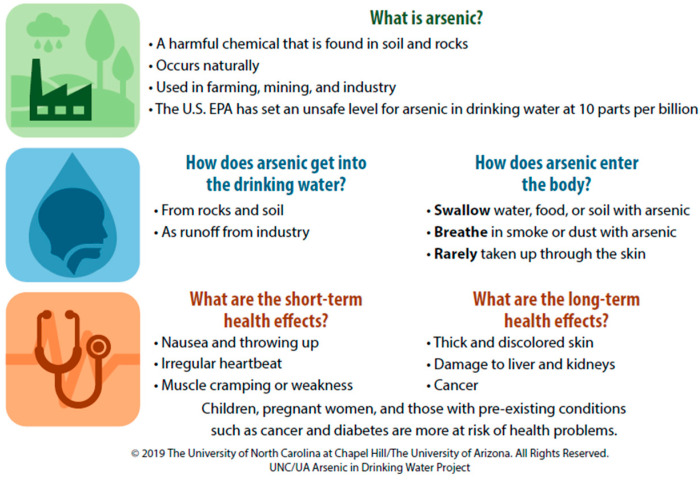
Arsenic and Health Handout.

During the research sessions, participants first completed self-efficacy surveys. Then they reviewed the family scenario and accompanying well test report (Figure 1) before responding to questions designed to provide insight into their ability to understand and interpret available information. After all participants had responded to the knowledge and skill questions, the second artifact—a handout with information about arsenic and health (Figure 2)—was distributed. Following distribution of this information, participants were allowed to revisit their initial answers and make changes, enabling collection of pre/post-assessment data on knowledge.

### 2.2. Data Collection

For this study, the revised WEHL was pilot tested with the two participant groups (i.e., undergraduate students majoring in non-STEM disciplines and well users in communities with toxic metals contamination). During each of four research sessions, participants individually completed surveys and responded to questions associated with the WEHL scenario. When all participants had completed the assessment, a focus group discussion elicited their perceptions of drinking water safety, their attitudes toward various sources of drinking water information, and their experience with WEHL, including perceived strengths, weaknesses, and any confusing aspects. Focus groups lasted between 30 and 60 min and were audio recorded. Recordings were transcribed verbatim by a transcription service.

Focus groups were chosen as the context for piloting WEHL because they are effective for formative assessment and enable participants to openly discuss their attitudes, beliefs, and perceptions. Group dynamics allow participants to compare their experiences, which can provide unique insights [24]. Additionally, this approach works well with groups of people who may be hesitant to share ideas in a one-on-one setting [25].

### 2.3. Data Sources and Analysis

Quantitative data sources included the following: (a) NVS scores, (b) WEHL scores, and (c) self-efficacy scores. The NVS scores were calculated using the NVS rubric, with scores ranging from 0–6 in increments of 1. The WEHL was scored using a rubric adapted from NVS, with scores ranging from 0–6 in increments of 0.5. Several WEHL questions were asked in a format that allowed participants to respond to secondary prompts (e.g., why or why not?), and partial credit (0.5 points) was awarded based on the accuracy of responses to these secondary prompts. This scoring approach enabled analysis of correlations between the NVS and WEHL results. Two WEHL questions regarding reliable information sources and trustworthy organizations were not scored because there were no parallel questions on the validated NVS. Notably, response to these questions varied widely. Self-efficacy scores were selected by participants, using a Likert scale that ranged from 1 (not at all certain) to 10 (highly certain).

Pearson’s correlation was completed to analyze correlations between NVS and WEHL scores. A Welch’s unequal variances t-test was used to compare the means of community resident and student NVS scores, as well as the means of the WEHL scores for each study group. An analysis of variance was completed to assess whether the WEHL score was affected by categorical demographic variables such as race, gender, and educational attainment. The analyses were performed on the data set as a whole and by group (community residents and undergraduate students). Chronbach’s alpha was used to assess the internal consistency of the adapted self-efficacy scale and the WEHL instrument. Data analysis for this paper was generated using SAS^®^ software, Version 9.4 of the SAS System for [Windows or Mac]. Copyright © 2015. SAS Institute Inc. SAS and all other SAS Institute Inc. product or service names are registered trademarks or trademarks of SAS Institute Inc., Cary, NC, USA.

Qualitative data were drawn from coded focus group transcripts, as well as WEHL artifacts that included participants’ written responses. Transcripts were coded by five members of the research team. Two coders participated in primary data collection while three others did not, thereby providing different perspectives to the coding process. Research team members initially coded one transcript individually using a priori codes with a mandate to add emergent codes. The team then met to discuss and reconcile codes into an initial codebook. Subsequently, the team met multiple times to review the process and iteratively revise the codebook. Differences in coding were reconciled through discussion.

## 3. Results

Results are presented below in two sections. The first section compares NVS (health literacy) and WEHL (environmental health literacy) scores. The second section presents data on various EHL components, including environmental health knowledge, skills, and self-efficacy scores.

### 3.1. Comparison of NVS and WEHL Scores

As described above, the health literacy score was calculated using the NVS, and the environmental health literacy score was calculated using the instrument piloted in this study. The NVS mean score across the entire sample (*n* = 47) was 4.77, which suggests adequate health literacy according to the NVS rubric [20] (see Table 1). The mean WEHL score across the sample was 4.12. There was a statistically significant (*p* < 0.0003), moderate degree of correlation between NVS and WEHL scores. Chronbach’s alpha was calculated for the 6-item WEHL instrument, with α = 0.61.

When the sample was split into groups (*n* = 24 undergraduate students, *n* = 23 community residents), student scores (for both NVS and WEHL), on average, were greater than 4 (Table 2). As above, this value suggests adequate health literacy using the NVS scoring rubric. Community residents’ scores on average were lower than 4, which could suggest more limited environmental health literacy. Additionally, using within-group analysis, we found a low degree of correlation between NVS scores and WEHL scores for students and a statistically significant (*p* < 0.0059) low-to-moderate degree of correlation for community residents (*p* < 0.01). For both NVS and WEHL scores by group, students scored significantly higher than residents (*p* < 0.0008 for NVs and *p* < 0.0132 for WEHL, see Table 3).

Across the sample, when EHL scores were analyzed by demographic variables, age was the only variable that generated significant results. Specifically, younger participants (≤24 years old, *n* = 23, 51%) scored significantly higher than older participants (>24 years old, *n* = 22, 49%) (younger group EHL mean = 4.61; older group EHL mean = 3.66; *p* = 0.02). Younger participants also scored significantly higher than older participants on NVS scores (younger group NVS mean = 5.61; older group NVS mean = 4.00; *p* = 0.0005). Using these age groupings, we were not able to cleanly separate students and community residents, since one student was older than 24, and one community resident was younger than 24. The age of 24 was used to sperate the groups because it was the median of the sample.

### 3.2. EHL Components: Environmental Health Knowledge

Participants’ environmental health knowledge was assessed by scoring responses to questions on the WEHL and analyzing coded comments from the focus groups.

#### 3.2.1. Responses to WEHL Questions

As noted above, the mean WEHL value for the entire sample was 4.12. Within groups, the student mean WEHL value was 4.63, and the community resident mean WEHL value was 3.59. In addition to computing these scores, correctness of responses was tabulated for each question (Table 4), using responses provided by participants before and after the arsenic and health handout was distributed. Overall, students tended to have a higher percentage of correct responses, but two questions proved challenging for both groups: question 2 (Is it safe for the Lee family to wash their hands with their tap water?) and question 3 (Why did the mother and daughter have health problems?).

For question 2, examples of incorrect responses included misunderstandings about routes of exposure, such as “contaminated water on their hands can be transferred easily to their mouth and be ingested” and that arsenic was easily absorbable through the skin. Correct responses more than doubled after the additional information was provided. However, a minority of respondents changed their answers from correct to incorrect, as with a student who initially answered this question correctly before receiving the arsenic handout and then changed their response, referencing symptoms of long-term arsenic ingestion. For question 3, most incorrect responses did not take into account the pre-existing conditions of the mother and daughter, such as in this example: “they had health problems because they consumed the water.”

#### 3.2.2. Focus Group Data on Environmental Health Knowledge and Skills

The student focus groups began with conversation about the sources of students’ drinking water in their home communities. (This question was not included in the community focus groups because community participants were screened into the study based on having private wells as the source of their drinking water.) Among students, knowledge of their drinking water sources varied; in each focus group, a subset of participants reported being unaware of the source.

Across all focus groups, participants identified a range of environmental health hazards in drinking water, with heavy metals (including arsenic) being the most commonly identified hazards. In the community focus groups, 24 unique participants identified nine heavy metals in addition to arsenic, which was introduced by researchers in the WEHL assessment scenario. These additional metals included (hexavalent) chromium, copper, lead, vanadium, and mercury, among others. In the student focus groups, however, lead was the only heavy metal other than arsenic that was mentioned. Importantly, the focus groups were conducted during and immediately following the high-profile Flint water crisis, which drew widespread attention to lead in water via media coverage. Within groups, student participants tended to refer to generic terms (such as “pollution”) to describe potential hazards while community participants identified categories of contaminants and specific chemicals within those categories (e.g., “biologicals” or “effluent” and “*E. coli*”).

In contrast, when discussing vulnerable populations, student participants identified more detailed and specific examples of vulnerable populations that might be exposed to water contamination, while community participants primarily referred to the vulnerable populations included in the WEHL assessment scenario. Student responses that went beyond the populations mentioned in WEHL included three unique mentions of low-income or socioeconomically disadvantaged populations and three unique mentions of people of color (including “Native Americans” and “Black and Brown communities”).

**Figure 3:**
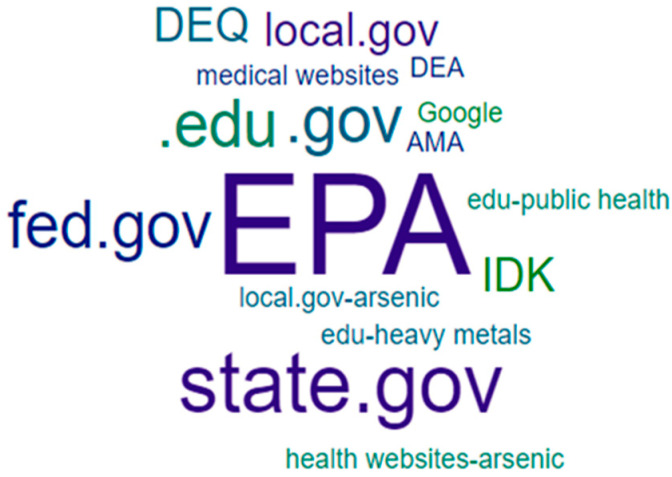
Community-identified sources of reliable information.

**Figure 4:**
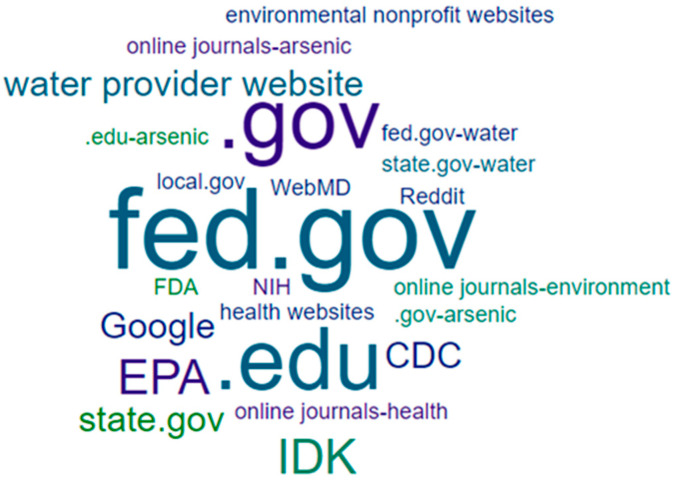
Student-identified sources of reliable information.

In response to questions about reliable information sources regarding well water safety, group responses also differed. For example, when participants were asked “if the family wanted additional information about arsenic, what types of websites would you suggest they review?”, they provided a wide range of responses that could be deemed correct. For this reason, this question was not scored nor was it included in the overall WEHL score. Instead, responses were aggregated based on broader, ad hoc categories of information sources. These categories were defined by two of the authors, with input and agreement from the others. A word cloud generator was used to create visualizations of responses (Figure 3 and Figure 4). As shown, students provided more responses overall and included research-oriented sources, while community residents correctly identified the agencies typically involved in well water issues in the state (local governments and the Department of Environmental Quality).

### 3.3. EHL Components: Self-Efficacy Scores

Across the sample (*n* = 47), mean responses to self-efficacy items ranged from 3.50 to 7.29, suggesting that participants’ self-efficacy varied from a level of “somewhat certain” that they could accomplish specific tasks to a moderately high level of certainty, depending on the question (Table 5). The lowest mean value was associated with the cost of testing, and the highest mean value was associated with sharing information with others. When the sample was split into groups, significant differences in self-efficacy were evident for items that incorporated distance (*p* < 0.0471) and cost (*p* < 0.0031 and *p* < 0.0225) (see Table 6), with students less likely to express confidence that they could accomplish testing when constrained by cost or distance. Chronbach’s alpha for the adapted 9-item self-efficacy scale was α = 0.88.

## 4. Discussion

As noted above, NVS and WEHL scores for the overall sample suggested adequate health literacy and EHL. When evaluated between groups, however, community residents’ scores were lower than those of the students and the overall sample, in a range that suggested the possibility of more limited EHL among community residents. Similar to findings reported by Irvin et al. [13], the statistically significant, moderate degree of correlation between mean NVS and mean WEHL scores suggested that if participants had adequate health literacy as assessed by NVS, they also were likely to have adequate EHL as assessed by the prototype instrument. However, when the two groups were analyzed separately, the correlation was no longer significant for students. In contrast, for community residents, the correlation was slightly stronger and was statistically significant, suggesting that NVS could be used as an initial assessment to understand the EHL of community residents, but it would not be effective for similar use with students.

These differences between the two groups raised questions about which variables may be influencing them, including the roles of demographics, environmental health knowledge, and perceptions of self-efficacy in participants’ scores. Although Irvin et al. [13] found positive associations between education and income level and EHL, analysis of demographic variables in this study did not yield significant findings, prompting analysis of participants’ responses to each of the items in WEHL and the self-efficacy survey. For WEHL, this analysis showed that participants in both groups incorrectly answered questions related to route of exposure and vulnerable populations. Although participant scores increased (overall and within groups) after they were provided with additional information on arsenic and health, fewer than half of all participants correctly answered these two questions. Notably, at the outset of the study a different version of the route-of-exposure question was flagged by environmental health professionals as potentially confusing. Although the question was revised, the number of incorrect responses make it difficult to know whether it was the concept or the wording of the question that was confusing to participants.

In open discussion during focus groups, additional differences emerged between community residents and students. Specifically, residents demonstrated a deeper knowledge of potential well water contaminants, which extended beyond the metals identified as concerns in their communities. They also were able to identify information sources with responsibility for well testing. Student participants identified a range of reliable information sources, but they were less aware of their drinking water sources and the agencies with relevant authority. They were, however, more attuned to populations that may be vulnerable to environmental health hazards. These differences underscored the ways that knowledge is contextualized and the role of lived experience in constructing knowledge. The student responses also raised questions for the research team about (a) whether the format of WEHL, which mimicked assessments used in formal instruction, may have influenced participants’ responses and (b) whether assessments that mimic tests may privilege people who are temporally closer to their formal educational experiences.

For the self-efficacy survey, across the entire sample participants felt moderately certain or better about their ability to complete many tasks. But when data were analyzed within groups, students’ and community residents’ responses differed in significant ways on several items: cost of well testing, distance to well testing, and cost of treatment. Notably, all were limiting factors for students, meaning that they felt less certain that they could accomplish the associated tasks when costs were higher or distances were farther. These responses are not surprising, given that students may have limited or variable income and limited transportation options, especially when traveling farther distances. Such limitations are a plausible explanation of the self-efficacy differences, though the small sample size complicates interpretation. Income and transportation constraints experienced by students may be similar to those of other low-income populations and suggest important areas of focus for educational and systemic interventions related to well water contamination (e.g., focusing on how well users could pay for or otherwise access testing and treatment resources).

As a reminder, the purpose of this study was to evaluate an instrument designed to help educators understand the baseline knowledge and self-efficacy of populations they engage. This information should inform the design of educational and other environmental health interventions to facilitate desired outcomes, especially when the interventions are envisioned as facilitating individual and collective action to protect health. The study’s purpose was not to encourage labeling of broad groups of people related to their literacy levels. One of the reasons for including self-efficacy items was to counter the deficit model often applied in projects focusing on public understanding of science [26], in which people are deemed to be science literate (or not) based on responses to a limited set of knowledge items. The authors argue that the field needs to move beyond the deficit model, to incorporate not only self-efficacy but also the influence of motivation and beliefs [27]. Assuming education serves as a means for growth and transformation, research may unnecessarily limit our understanding of such development and associated learning outcomes when it focuses only on knowledge as determined by correct responses to test questions.

In summary, the WEHL provided a context for exploring essential environmental health concepts (e.g., hazard, exposure routes, vulnerable populations) and self-efficacy associated with documenting and remediating well water contamination. Although students scored higher than community residents on both process-focused literacy instruments, most participants across the entire sample could decide appropriately whether to drink the arsenic-contaminated water. Many were challenged to know whether the water was safe for other uses, and some were effective at quickly integrating new information into their decision-making processes. Important limiting criteria for decision-making also were identified, and WEHL provided insights into the knowledge and skills participants relied on to make these decisions (for instance, how they incorporated information about exceedance of federal standards), how their lived experiences differed, and where each group might seek information to expand their knowledge and skills.

### Limitations

The limitations of this study included small sample size and limited racial/ethnic diversity of the sample; and these limitations mean that the findings are not generalizable. Members of the research team from the University of Arizona expect to engage more diverse populations in future research sessions. A greater number and diversity of participants may not only offset these limitations but also may help to confirm initial findings. Another limitation was voluntary participation, because those who chose to participate may have differed in important ways from those who did not. Finally, participant responses were limited to a single point in time, preventing assessment of change over time in the measured parameters.

## 5. Conclusions

These findings suggest that WEHL can be used as a starting point for educators who wish to understand learners’ knowledge and self-efficacy in the context of well water contamination. However, this analysis raises questions about how well understood some essential environmental health concepts are, as well as the kinds of skills development and other factors that may enhance EHL associated with well water contamination. Further research on how context-specific skills and self-efficacy contribute to EHL is needed. Additionally, further research with more diverse audiences should provide insight into the general applicability of these findings and the extent to which sociodemographic variables may influence EHL.

Given that WEHL did not capture the varied contextual knowledge of some participants (which instead emerged during discussion afterwards), this analysis raises questions about whether tools that mimic assessments from formal education are adequate for populations that are temporally removed from formal education. For this reason, it is recommended that future research on EHL include methods that feel less like test-taking and also provide opportunities to understand participants’ motivation and relevant attitudes.

## Figures and Tables

**Table 1:** NVS and WEHL scores (*n* = 47).

Variable	Mean	Std Dev	Range	Pearson Correlation Coefficient	*p*-Value
NVS score (health literacy)	4.77	1.71	0–6	**0.5076**	0.0003 *
WEHL score (env. health literacy)	4.12	1.46	1–6		

* Significant values are bolded.

**Table 2:** NVS and WEHL scores for groups.

Group	Variable	Mean	Std Dev	Range	Pearson Correlation Coefficient	*p*-Value
Undergraduate Students (*n* = 24)	NVS score (health literacy)	5.58	0.78	3–6	0.0117	0.9568
	WEHL score (env. health literacy)	4.63	1.20	1.5–6		
Community Resident (*n* = 23)	NVS score (health literacy)	3.91	2.00	0–6	**0.5557**	0.0059 *
	WEHL score (env. health literacy)	3.59	1.54	1–6		

* Significant values are bolded.

**Table 3:** Comparison of group NVS and WEHL scores.

Group	NVS Mean	*p*-Value	WEHL Mean	*p*-Value
Undergraduate Students (*n* = 24)	5.58	**0.0008**	4.63	0.0132 *
Community Residents (*n* = 23)	3.91		3.59	

* Significant values are bolded.

**Table 4:** Correct responses to environmental health knowledge questions.

WEHL Question	Total, *n* = 47		Students, *n* = 24		Community, *n* = 23	
	Number of Correct Response (%)					
	Pre	Post	Pre	Post	Pre	Post
Q1. Is it safe for the family to drink their tap water? Why or why not?	37 (78.7)	38 (80.9)	23 (95.8)	23 (95.8)	14 (60.9)	16 (69.6)
Q2. Is it safe for the family to wash their hands with their tap water? Why or why not?	8 (17.0)	21 (44.7)	4 (16.7)	11 (45.8)	4 (17.4)	10 (43.5)
Q3. Why did the mother and daughter have health problems?	9 (19.1)	21 (44.7)	7 (29.2)	16 (66.7)	2 (8.7)	5 (21.7)
Q4. Would their health be affected if they continued to drink their tap water for many years? Why or why not?	24 (51.1)	32 (68.1)	15 (62.5)	18 (75.0)	9 (39.1)	14 (60.9)
Q5. In how many homes in the neighborhood is the water safe to drink?	39 (83.0)	39 (83.0)	22 (91.7)	22 (91.7)	17 (73.9)	17 (73.9)
Q6. What is the difference between the unsafe level of arsenic set by the federal government and the amount in their tap water?	30 (63.8)	32 (68.1)	15 (62.5)	17 (70.8)	15 (65.2)	15 (65.2)

**Table 5:** Responses to self-efficacy questions.

	*n*	Mean
1. I can learn whether my well water contains arsenic…		
If a water testing facility is nearby (within a one-hour drive).	46	5.46 ± 3.43
If a water testing facility is far away (more than a one-hour drive).	46	4.04 ± 3.56
If a water test costs $50 or less.	45	4.78 ± 3.93
If a water test costs more than $50.	46	3.50 ± 3.32
2. I can find someone to test my well water for arsenic.	46	5.02 ± 3.42
3. I can find reliable information about any risks of arsenic in well water.	47	6.57 ± 3.18
4. I can share with others the information I learn about any risks of arsenic in well water.	45	7.29 ± 3.12
5. I can do the kinds of things needed to remove arsenic from my well water…		
If the recommended treatment costs $100 or less.	45	4.93 ± 3.41
If the recommended treatment costs more than $100.	45	3.91 ± 3.34

**Table 6:** Self-Efficacy items with statistically significant differences between groups.

Self-Efficacy Questions	Undergraduate Students			Community Residents			*p*-Value *
	*n*	Mean ± Std Dev	Range	*n*	Mean ± Std Dev	Range	
I can learn whether my well water contains arsenic…							
If a water testing facility is nearby.	24	4.50 ± 3.30	0–10	22	6.50 ± 3.33	0–10	**0.0471**
If a water test costs $50 or less.	24	3.54 ± 3.30	0–9	21	6.19 ± 4.19	0–10	**0.0031**
If a water test costs more than $50.	24	2.13 ± 2.40	0–9	22	5.00 ± 3.59	0–10	**0.0031**
I can do the kinds of things needed to remove arsenic from my well water…							
If treatment costs more than $100.	24	2.83 ± 2.58	0–8	21	5.14 ± 3.72	0–10	**0.0225**

* All *p*-values represent significant differences between student and community responses using Welch’s unequal variance *t*-test.

## Data Availability

The data presented in this study are available on request from the corresponding author.

## Appendix A

**Table A1:** Demographic characteristics of study participants.

	Students, *n* = 24 (51.1%)	Community Residents, *n* = 23 (48.9%)	Total Sample *n* = 47
Gender			
Female	16 (66.6)	11 (47.8)	27 (57.4)
Male	7 (29.2)	12 (52.2%)	19 (40.4)
Non-binary	1 (4.2)	-	1 (2.1)
Age (yr) (*n* = 43)			
Median	21	68	24
Range	18–26	26–77	18–77
Race/Ethnicity			
White	9 (37.5)	18 (78.7)	27 (57.4)
Black or African American	2 (8.3)	4 (17.4)	6 (12.8)
Hispanic, Latino or Spanish Origin	7 (29.2)	-	7 (14.9)
Other	6 (25.0)	1 (4.4)	7 (14.9)
Education (*n* = 46)			
High school graduate or some college, no degree	20 (83.3)	5 (22.7)	25 (54.3)
Associate’s degree/Bachelor’s degree	4 (16.7)	9 (40.9)	13 (28.2)
Master’s degree or higher	-	8 (34.8)	8 (17.4)
Developed Environment (*n* = 45)			
Urban/Suburban	17 (73.9)	2 (0.09)	19 (42.2)
Rural	6 (26.1)	20 (90.1)	26 (57.8)
